# Safety and durability of the immune response after vaccination with the heterologous schedule of anti-COVID-19 vaccines SOBERANA®02 and SOBERANA® Plus in children 3–18 years old

**DOI:** 10.1016/j.jvacx.2024.100595

**Published:** 2024-12-06

**Authors:** Dagmar García-Rivera, Rinaldo Puga-Gómez, Sonsire Fernández-Castillo, Beatriz Paredes-Moreno, Yariset Ricardo-Delgado, Meiby Rodríguez-González, Carmen Valenzuela Silva, Rocmira Pérez-Nicado, Laura Rodríguez-Noda, Darielys Santana-Mederos, Yanet Climent-Ruiz, Enrique Noa-Romero, Otto Cruz-Sui, Belinda Sánchez-Ramírez, Tays Hernández-García, Ariel Palenzuela-Diaz, Marisel Martínez-Perez, Arilia García-López, Yury Valdés-Balbín, Vicente G. Vérez-Bencomo

**Affiliations:** a Finlay Vaccine Institute, Av. 21 #19810, Atabey, Playa, Havana 11600, Cuba; b Pediatric Hospital “Juan Manuel Márquez”. Av. 31, Marianao, Havana 11400, Cuba; c National Civil Defense Research Laboratory. San José de Las Lajas, Mayabeque 32700, Cuba; d Center for Molecular Immunology. Av 15th. and 216 Street, Siboney, Playa, Havana 11600, Cuba; e Center for Immunoassays. 134 St and 25, Cubanacán, Playa, La Habana 11600, Cuba; f National Clinical Trials Coordinating Center. Av. 5th and 62 Street, Miramar, Playa, Havana 11300, Cuba

**Keywords:** COVID-19 vaccine, RBD, Cellular response, Pediatric vaccine, Conjugate vaccine, Subunit vaccine, SARS-CoV-2

## Abstract

**Background:**

The heterologous three-dose schedule of the protein subunit anti-COVID-19 SOBERANA®02 and SOBERANA® Plus vaccines has proved its safety, immunogenicity and efficacy in pediatric population, but durability of immunogenicity is not yet dilucidated. This study reports the safety and durability of the humoral and cellular responses in children and adolescents 5–7 months after receiving the heterologous vaccine schedule of SOBERANA® 02 and SOBERANA® Plus.

**Methods:**

Children participating in a phase I/II clinical trial were followed-up for 5–7 months after the last dose. They were clinically examined by medical doctors, and their parents were interviewed searching for long-term adverse events. Blood samples were collected to evaluate the duration of humoral and cellular immune responses. Sera were tested for the presence of SARS-CoV-2 nucleocapsid (N) protein.

**Results:**

There were no reports of severe adverse events such as coagulation disorders, myocarditis, or pericarditis. None of the participants who withdrew from the trial during the follow-up period did so due to post-vaccination adverse events. The humoral response waned in time for N-negative children, but levels of specific and neutralizing antibodies remained similar to those attained after the second dose of SOBERANA® 02 in the heterologous schedule. Neutralizing antibodies against SARS-CoV-2 D614G and omicron BA.1 were detected 5–7 months post-vaccination. RBD-specific IFN-γ secreting cells showed no significant change compared to levels following primary immunization, in both N-negative and N-positive children.

**Conclusions:**

The vaccination regimen was safe over time, and both humoral and cellular immunity persisted in the vaccinated population aged 3–18 years, 5–7 months after receiving the heterologous SOBERANA® 02 and SOBERANA® Plus vaccine schedule.

Trial registry: https://rpcec.sld.cu/trials/RPCEC00000374-En

## Highlights

• Pediatric vaccination with SOBERANA® 02 and SOBERANA® Plus was safe over time.
• Humoral and cellular immune response is sustainable until 5–7 months after vaccination.
• Neutralizing antibodies detected 5–7 months after vaccination are cross-reactive to omicron BA.1 strain.

## 1 Introduction

COVID-19 vaccination in pediatric population has been associated with the reduction of disease incidence and hospitalizations among this age group, contributing to the pandemic's control [[1], [2], [3]]. Currently, healthy children and adolescents aged 6 months to 17 y/o has been classified into the low priority- vaccine use group according to the WHO roadmap in the context of omicron circulation and high population immunity [4]. However, the emergence of a new SARS-CoV-2 variant with increased pathogenicity is still a potential risk, therefore, the continued research of vaccine effects on all populations remains relevant.

The heterologous vaccination regimen of two doses of SOBERANA®02 followed by one dose of SOBERANA® Plus, administered 28 days apart, was used for pediatric immunization in Cuba and in some other countries during the COVID-19 pandemic [[5], [6], [7]]. This vaccination scheme has been incorporated into the routine immunization schedule for children at 2 years of age in the post-pandemic era in Cuba. During the clinical development, this scheme demonstrated its safety, immunogenicity and efficacy in both adults and pediatric population [[8], [9], [10], [11]]. Particularly, in children and adolescents aged 3–18 years, this vaccination schedule showed a very low frequency of systemic adverse events, an effective virus neutralization against various variants of concern (including omicron BA.1), a neutralizing response comparable to that in young adults, a robust T-cell response, and the induction of long-term immunological memory [11,12].

The durability of antibody response after primary vaccination has been studied in recipients of mRNA vaccines [[13], [14], [15]]; there is less data available on the durability of the immune responses to protein subunit vaccines. The present study examines the safety and durability of the humoral and cellular responses in children who received the heterologous scheme of SOBERANA® 02 and SOBERANA® Plus protein subunit vaccines in a 5–7-month follow-up period as part of the phase I/II clinical trial.

## 2 Materials and methods

### 2.1 Study design and approvals

A phase I/II open-label, multicenter, and adaptive study was designed to evaluate the safety, reactogenicity, and immunogenicity of the primary vaccination consisting of two doses of SOBERANA®02 (SARS-CoV-2 recombinant receptor-binding domain, RBD, chemically conjugated to tetanus toxoid adjuvanted in alumina) and a third dose of SOBERANA® Plus (recombinant dimeric RBD adjuvanted in alumina) administered 28 days apart in children 3–18 y/o (herein stage 1). The safety and immunogenicity results of this primary immunization are published [11].

In January 2022, the Ethical Committee of the “Juan Manuel Marquez” Pediatric Hospital and the National Regulatory Agency CECMED approved a modification of this clinical trial (trial registry: https://rpcec.sld.cu/trials/RPCEC00000374-En) to evaluate the safety and immunogenicity in participants after a follow-up period of 5–7 months (herein stage 2).

### 2.2 Stage 2. Subjects and ethics

The study population was the 306 subjects that completed the three doses schedule during the stage 1. During recruitment the medical doctors provided to the parents, both orally and written, all information about this new stage of the trial. A new signed informed consent was requested to the parents and also, it was requested the assent of the adolescents (participants 12–18 years-old). The trial was conducted according to Helsinki's Declaration, Good Clinical Practice and the Cuban National Immunization Program requirements. Children were excluded of the follow-up only if the received other COVID-19 vaccine during this period.

### 2.3 Safety assessment

The children were called for a follow-up visit 5–7 months after the last dose (SOBERANA® Plus) of the heterologous vaccination schedule (stage 1). They were clinically examined by medical doctors, and their parents were interviewed searching for long-term adverse events that might be related to vaccination. Children whose parents reported having suffered from COVID-19 (confirmed by RT-PCR SARS-CoV-2) during the follow-up period were considered in the safety and immunogenicity analysis in stage 2.

### 2.4 Immunogenicity assessment

Blood samples from all participants were collected for evaluating the duration of humoral immune response. Peripheral blood mononuclear cells (PBMCs) were isolated from a subset of 14 children randomly selected for evaluating the T cell response six months after primary vaccination.

Anti-RBD IgG was determined by a quantitative ultramicro enzyme-linked immunoassay (UMELISA SARS-CoV-2 anti- RBD). The capacity of anti-RBD antibodies for blocking the RBD-hACE2 interaction was determined by a competitive ELISA and expressed as percentage inhibition and molecular virus neutralization titer (mVNT_50_). The conventional virus neutralization titer was determined in a subset of samples randomly selected by the live-virus assay (cVNT_50_) vs D614G (CU2010–2025, hCoV-19/Cuba/DC01/2020/ GIDAID: EPI_ISL_7495115|2020-06-05) and omicron (BA1.21 K, RRR, hCoV-19/Cuba/DC-RRR/2201/ GISAID: EPI_ISL_12691753|2022-05-15) variants. RBD-specific T cells producing IFN- γ and IL- 4 were estimated by ELISPOT. These immunological techniques were as previously described [11].

### 2.5 Detection of anti- SARS-CoV-2 nucleocapsid (N) protein antibody in serum samples by ELISA

All serum samples were tested for SARS-CoV-2 nucleocapsid (N) protein in the search for asymptomatic infections during the follow-up period. For data analysis the population under study was subdivided into N-positive and N-negative children.

Detection of SARS-CoV-2 nucleocapsid (N) protein in serum samples was performed by the UMELISA protein N assay (CIE, Immunoassay Center, Havana, Cuba). Briefly, 10 μl of a 1/20 dilution of serum in Tris 0,371 mol/L-sheep serum 5 % were added to ELISA plates (Greiner Bio-one, Germany) coated with recombinant SARS-CoV-2 nucleocapsid protein (CIGB, Cuba). Plates were incubated for 30 min at 37 °C in a humid chamber. After washing the plates, 10 μl of alkaline phosphatase anti-human IgG in Tris 0,05 mol/L + Tween 20 0,05 % + BSA 1 % were added to each well and incubated as previously. After a wash step, 10 μl/well of 4-methylumbelliferyl phosphate substrate was added. Fluorescence was read in a SUMA reader (CIE, Immunoassay Center, Havana, Cuba) after 30 min incubation in the dark. Samples with a fluorescent value higher than 30 were considered as positive; this cut off value was calculated as the mean of the negative control samples plus 3 standard deviations.

### 2.6 Data management and statistical analysis

We utilized the “OpenClinic” medical record system (http://openclinic.sourceforge.net) to electronically store all data.

Safety and reactogenicity were expressed as frequencies (%). Anti-RBD IgG concentration and % of inhibition of RBD-hACE2 interaction were expressed as median and interquartile range; molecular virus neutralization titer (mVNT50) and conventional virus neutralization titer (cVNT50) were expressed as geometric mean (GMT) and 95 % confidence intervals (CI). The Wilcoxon signed-rank test was used for before-after statistical comparison and Mann-Whitney *U* test was used for comparison between N-positive and N-negative individuals.

Statistical analyses were done using SPSS version 25.0; EPIDAT version 12.0 and Prism GraphPad version 6.0. An alpha signification level of 0.05 was used.

## 3 Results

### 3.1 Demographic characteristic of subjects and flow chart

**Fig. 1:**
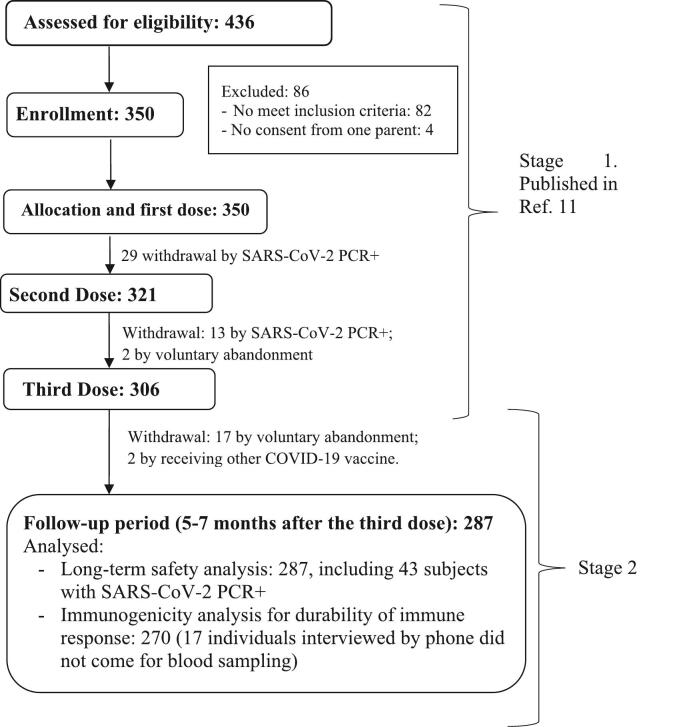
Flow chart. Stage 1: Phase I/II clinical trial: recruitment, inclusion and vaccination with the heterologous schedule of two doses of SOBERANA®02 and a heterologous third dose of SOBERANA® Plus 28 days apart of children aged 3–18 years old. Stage 2: follow-up for safety and durability of the immune response 5–7 months after the third dose.

From January to April 2022 (5–7 months after the last dose of the primary schedule), the 306 children that had completed the heterologous vaccination scheme (stage 1) were called for a follow-up consultation. Of them, 19 were excluded because parents refused for participating (*n* = 17) or because children received another COVID-19 vaccine (*n* = 2). During the follow-up period, 43 children were positive to RT-PCR SARS-CoV-2; they were included in the safety and immunogenicity analysis (Fig. 1). For immunogenicity analysis, 17 individuals that were interviewed by phone call did not came for blood sampling. Table 1 shows the demographic characteristics of 287 children included in stage 2.

**Table 1:** Demographic characteristics of subjects included in follow-up study (stage 2).

	Age groups		
	3–11 years	12–18 years	Total
**N**	147	140	287
**Sex**			
Female	69 (46.9 %)	65 (46.4 %)	134 (46.7 %)
Male	78 (53.1 %)	75 (53.6 %)	153 (53.3 %)
**Skin color**			
White	110 (74.8 %)	94 (67.1 %)	204 (71.1 %)
Black	8 (5.4 %)	10 (7.1 %)	18 (6.3 %)
Multiracial	29 (19.7 %)	36 (25.7 %)	65 (22.6 %)
**Age (years)**			
Mean (SD)	7.4 (2.5)	14.9 (2.1)	11.11 (4.4)
Median (IQR)	8.0 (5.0)	15.0 (4.0)	11.0 (7.0)
Range	3; 11	12;18	3;18
**Weight (kg)**			
Mean (SD)	29.2 (9.9)	54.4 (9.2)	41.5 (15.8)
Median (IQR)	27.5 (14.0)	55.0 (13.4)	42.0 (28.0)
Range	13.0; 55.0	32.0; 80.0	13.0; 80.0
**Height (cm)**			
Mean (SD)	128.8 (17.3)	164.3 (9.7)	146.1 (22.7)
Median (IQR)	131.0 (26.0)	163.5 (14.0)	151.0 (33.0)
Range	92; 169	142; 190	92–190
**BMI (kg/m**^**2**^**)**			
Mean (SD)	17.0 (2.0)	20.2 (2.4)	18.5 (2.6)
Median (IQR)	16.7 (2.7)	19.9 (3.9)	18.2 (3.7)
Range	13.6; 22.8	14.6; 25.3	13.6; 25.3

Data are n (%) unless otherwise specified. Mean (SD) = Mean ± Standard Deviation. Median (IQR) = Median ± Interquartile Range. BMI=Body mass index. Range = (Minimum; Maximum).

### 3.2 Long term safety of primary vaccination with the heterologous scheme

The parents of 287 children were extensively interviewed to identify potential long-term adverse events related to the vaccination. There were no reports of special adverse events like coagulation disorders, myocarditis, or pericarditis. None of the participants who withdrew from the study during the follow-up period did so due to post-vaccination adverse events. Among the 43 RT-PCR SARS-CoV-2 positive children, there were no reports of severe COVID-19 nor multisystemic inflammatory syndrome.

### 3.3 Antibody response 5–7 months after the primary heterologous scheme

Table 2 summarizes the immunogenicity data in 270 vaccinated children, 5–7 months after the last (heterologous) dose in stage 1. Children were divided in two subgroups according to the presence of protein N in serum.

**Table 2:** Durability of the antibody response in children, determined 5–7 months after the heterologous vaccination schedule of two doses of SOBERANA®02 followed by one dose of SOBERANA® Plus.

**Follow-up period:** Min: 5 months Max: 7 months		Age group			Age sub-group			Age sub-group		
		3–18 years-old			3–11 years-old			12–18 years-old		
		Protein N positive	Protein N Negative	*P*-value	Protein N positive	Protein N Negative	P-value	Protein N positive	Protein N Negative	P-value
N		201	69		100	38		101	31	
IgG anti-RBD (UA/mL)	Median	213.1	80.9	<0.001⁎	241.8	93.4	<0.001⁎	164.5	57.6	<0.001⁎
	25th-75th	104.3; 477.3	36.0; 179.4		133.4; 510.9	39.5; 226.8		88.1; 416.7	35.9; 145.5	
% Inh RBD:hACE2	Median	92.9	86.3	<0.001⁎	93.8	90.2	<0.001⁎	91.4	83.0	<0.001⁎
	25th-75th	90.5; 94.2	60.2; 92.0		92.2; 95.1	66.9; 94.2		88.6; 93.3	55.1; 89.1	
mVNT_50_	GMT	1490.5	428.4	<0.001⁎	1872.0	661.7	0.001⁎	1189.4	251.4	<0.001⁎
	CI 95 %	1264.7; 1756.5	287.4; 638.6		1466.7; 2389.4	378.2; 1157.9		959.1; 1448.9	146.9; 430.5	
N		92	56		19	29		73	27	
cVNT_50_ vs D614G	GMT	302.0	61.1	<0.001⁎	360.9	68.9	<0.001⁎	288.3	53.7	<0.001⁎
	CI 95 %	232.0; 393.0	42.0; 89.0		210.4; 619.0	41.2; 115.4		212.2; 391.6	30.1; 95.9	
N		30	36		2	26		28	10	
cVNT_50_ vs omicron BA.1	GMT	138.6	37.1	<0.001⁎	320.0	40.1	0.037⁎	130.5	30.2	0.001⁎
	CI 95 %	92.9; 206.6	24.4; 56.7		160; 640.0^R^	23.7; 67.8		86.3; 197.4	13.6; 67.1	

IgG anti-RBD: Concentration of IgG anti-RBD antibodies, UA/mL = arbitrary units/mL. % Inh RBD:hACE2: Percentage of inhibition of RBD:hACE2 interaction at 1/100 serum dilution. mVNT50: Molecular neutralization titer. cVNT50: Viral neutralization titer. GMT = Media Geometric titer. CI 95 % = Confidence Interval 95 %; 25th–75th = percentile 25–75.

⁎ *p* < 0.005 Mann-Whitney U test (IgG anti-RBD, % Inh RBD:hACE2) or Student's *t*-Test (mVNT_50_, cVNT_50_, log-transformed). N: represent the total children with every determination available.

We found a remarkable anti-SARS-COV-2 immune response 5–7 months after vaccination in the 69 N-negative children: the median of anti-RBD IgG concentration was 80.9 AU/mL (25–75 percentile, 36.0, 179.4), the inhibition of RBD-ACE2 interaction was 86.3 % (25–75 percentile, 60.2, 92.0), and the molecular neutralization titer (mVNT_50_) was 424.4 (CI 95 % 287.4; 638.6). Viral neutralization persisted over time with titers against D614G of 61.1 (CI 95 % 42.0; 89.0) and 37.1 (CI95 % 24.4; 56.7) against the omicron BA.1 variant. Immune responses generally appeared greater among children aged 3–11 than those with 12–18 y/o, despite of N-positive or N-negative status (Table 2).

As expected for hybrid immunity, the immune response in N-positive children was significantly higher than in N-negative children in both age subgroups. In N-positive children anti-RBD IgG was 213.1 UA/mL (25–75 percentile, 104.3, 477.3), mVNT_50_ was 1490.5 (CI 95 % 1264.7; 1756.5), cVNT_50_ vs D614G was 302.0 (CI 95 % 232.0; 393.0) and was 138.6 (CI 95 % 92.9; 206.6) vs omicron BA.1 (Table 2).

### 3.4 Kinetics of immune response after the vaccination and during the follow-up period

**Fig. 2:**
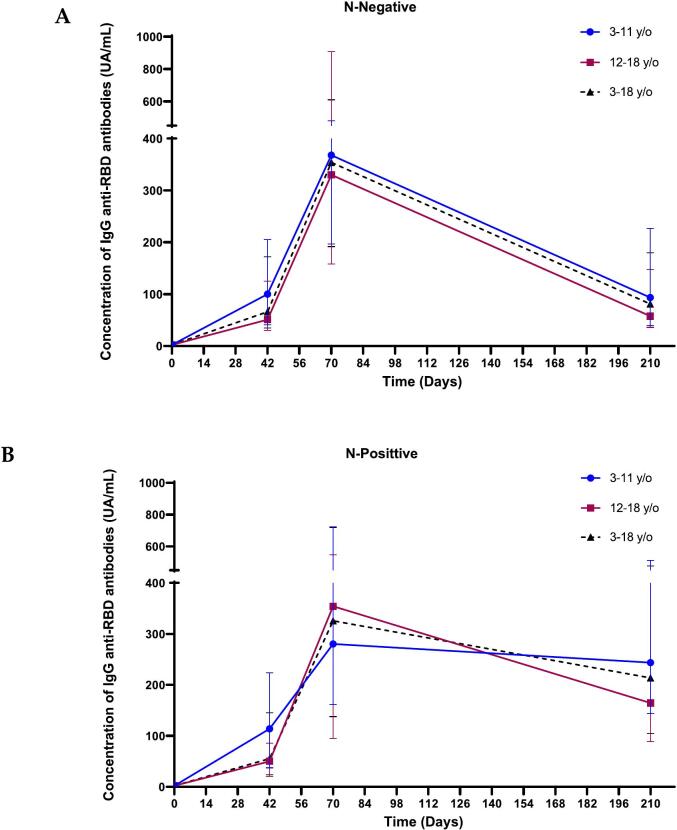
Kinetics of IgG anti-RBD in N-Negative (A) and N-Positive (B) children. Children received two doses of SOBERANA® Plus (T0 and T28) and a third dose with SOBERANA®Plus. Anti-RBD IgG was measured on T0 (before immunization), T42 (14 days after second immunization), T70 (14 days after third immunization) and between 5 and 7 months after the last dose (represented as T210 in the graph). The Wilcoxon signed-Rank Test was used for before-after statistical comparison.

The kinetics of IgG anti-RBD antibody response in N-Negative children shows a maximum after the third dose of the heterologous schedule. As expected, IgG anti-RBD decreases over time. After 5–7 months they attain values similar to those elicited after the second dose of SOBERANA® 02 (received at least 6 months earlier) (Fig. 2A). However, the antibody neutralization capacity measured by molecular and conventional neutralizing assays is still significantly higher (*p* < 0.05) than those measured after the second dose (Table 3). Compared to the immune response elicited after the third dose, the N-positive children have similar anti-RBD IgG and mVNT_50_ values, and higher % inhibition and cVNT_50_ values 5–7 months after completing the immunization schedule, as the exposure to the virus acts as an immunological booster (Table 4 and Fig. 2B).

**Table 3:** Kinetics of neutralizing immune response in N-negative children.

Follow-up period: Min: 5 months Max: 7 months after the third dose		Age group			Age sub-group					
		3–18 years-old			3–11 years-old			12–18 years-old		
		Protein N Negative			Protein N Negative			Protein N Negative		
		Post-2nd dose	Post-3rd dose	5–7 months	Post-2nd dose	Post-3rd dose	5–7 months	Post-2nd dose	Post-3rd dose	5–7 months
N		69	69	69	38	38	38	31	31	31
% Inh RBD: hACE2	Median	71.5	92.9	86.3^⁎, ⁎⁎^	77.7	92.5	90.2^⁎, ⁎^	61.9	93.0	83.0 ^N·S.,⁎⁎^
	25th–75th	48.0; 87.2	89.4; 93.6	60.2; 92.0	49.8; 87.5	88.6; 93.3	66.9; 94.2	43.1; 84.4	90.0; 93.9	55.1; 89.1
mVNT_50_	GMT	238.4	1505.0	428.4^⁎, ⁎⁎^	286.6	1461.3	661.7^⁎, ⁎^	190.2	1560.3	251.4 ^N·S.,⁎⁎^
	CI 95 %	168.0; 338.3	1102.7; 2054.0	287.4; 638.5	177.1; 463.7	974.7; 2191.0	378.2; 1157.9	112.2; 322.4	938.0; 2595.4	146.9; 430.5
N		22	22	22	10	10	10	12	12	12
cVNT_50_ vs D614G	GMT	32.2	225.2	52.0^NS, ⁎⁎^	28.6	138.7	37.2^N·S, ⁎^	35.6	337.2	68.2^NS, ⁎⁎^
	CI 95 %	14.5; 71.6	107.0; 473.9	31.4; 86.0	5.8; 139.7	32.0; 600.7	16.0; 86.1	14.0; 90.3	152.8; 743.9	34.9; 135.8

% Inh RBD:hACE2: Percentage of inhibition of RBD:hACE2 interaction at 1/100 serum dilution. mVNT_50_: Molecular neutralization titer. cVNT_50_: Viral neutralization titer. MGT = Media Geometric titer. CI 95 % = Confidence Interval 95 %. N: represent the total children with every determination available.

*,* *p* < 0.05Wilcoxon test (% Inh RBD:hACE2) or Student's t-Test (mVNT_50_, cVNT_50_ log-transformed) vs. Post 2nd dose (first *), vs. post 3rd dose (second *).

**,**p < 0.005 Wilcoxon test (% Inh RBD:hACE2) or Student's t-Test (mVNT_50_, cVNT_50_ log-transformed) vs. Post 2nd dose (first **), vs. post 3rd dose (second**).

**Table 4:** Kinetics of neutralizing immune response in N-positive children.

Follow-up period: Min: 5 months Max: 7 months after the third dose		Age group			Age sub-group					
		3–18 years-old			3–11 years-old			12–18 years-old		
		Protein N Positive			Protein N Positive			Protein N Positive		
		Post-2nd dose	Post-3rd dose	5–7 months	Post-2nd dose	Post-3rd dose	5–7 months	Post-2nd dose	Post-3rd dose	5–7 months
N		200	200	200	99	99	99	101	101	101
% Inh RBD:hACE2	Median	68.4	92.1	92.9^⁎⁎, ⁎⁎^	80.7	92.5	93.8^⁎⁎, ⁎⁎^	60.2	91.7	91.4^**⁎⁎.** N.S^
	25th–75th	40.3; 86.8	87.6; 93.5	90.5; 94.2	45.6; 91.1	88.7; 93.5	92.2; 95.1	33.6; 77.7	86.6; 93.5	88.6; 93.3
mVNT_50_	GMT	193.5	1255.9	1506.3^**⁎⁎.** N.S^	284.2	1458.0	1916.9^⁎⁎, NS^	132.8	1085.0	1189.4 ^**⁎⁎.** N.S.^
	CI 95 %	156.4; 239.5	1072.2; 1471.1	1278.8; 1774.3	209.9; 384.8	1161.0; 1830.9	1505.0; 2441.5	99.8; 176.8	871.1; 1351.6	959.1; 1474.9
N		49	49	49	16	16	16	33	33	33
cVNT_50_ vs D614G	GMT	20.8	126.2	388.8^⁎⁎, ⁎⁎^	45.8	215.8	459.0^**⁎⁎.** N.S.^	14.2	97.3	358.7^⁎⁎, ⁎⁎^
	CI 95 %	13.6; 31.6	90.3; 176.5	261.3; 578.4	18.1; 115.5	112.5; 413.8	264.2; 797.6	9.4; 21.3	66.8; 141.8	208.4; 617.3

% Inh RBD:hACE2: Percentage of inhibition of RBD:hACE2 interaction at 1/100 serum dilution. mVNT_50_: Molecular neutralization titer. cVNT_50_: Viral neutralization titer. MGT = Media Geometric titer. CI 95 % = Confidence Interval 95 % N: represent the total children with every determination available.

*,*p < 0.05Wilcoxon test (% Inh RBD:hACE2) or Student's t-Test (mVNT_50_, cVNT_50_ log-transformed) vs. Post 2nd dose (first *), vs. post 3rd dose (second *).

**,**p < 0.005 Wilcoxon test (% Inh RBD:hACE2) or Student's t-Test (mVNT_50_, cVNT_50_ log-transformed) vs. Post 2nd dose (first **), vs. post 3rd dose (second**).

**Fig. 3:**
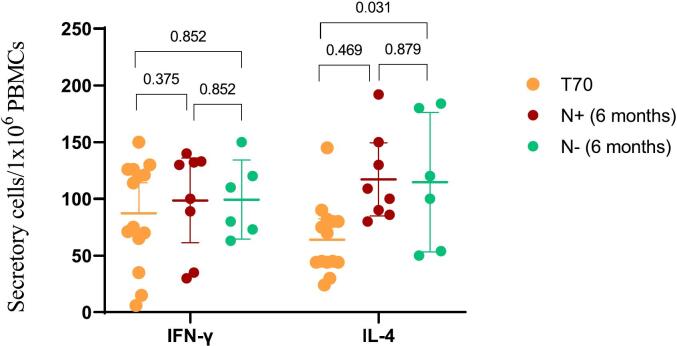
RBD-specific Interferon-γ (INF-γ) and interleukin-4 (IL-4) secreting cells in peripheral blood mononuclear cells (PBMCs) in 14 randomly selected subjects aged 3–18 y/o. PBMC were obtained fourteen days after the third dose of the heterologous schedule with SOBERANA®02 and SOBERANA® Plus vaccines (T70) and 6 months after schedule conclusion (paired samples). At the follow-up period, samples were classified as N-positive (N+) or N-negative (N-) according to SARS-CoV-2 nucleocapsid protein ELISA assay. All samples were N-negative at T70. Wilcoxon signed-rank test was used for before-after statistical comparison and Mann-Whitney *U* test for N+ vs N- comparison.

PBMCs from a subset of 14 randomly selected children were analyzed for RBD-specific T-cells producing IFN-γ and IL-4 six months after vaccination. Results were compared to paired samples obtained 14 days after the third dose of the heterologous schedule (T70). As shown in Fig. 3, six months after vaccination there were no statistical differences in the number of RBD-specific IFN-γ secreting T cells compared those attained at T70, in both N-positive and N-negative individuals. The number of RBD-specific IL-4 secreting T cells was higher (*p* = 0.031) at the follow up period in N-negative individuals compared to T70. Additionally, for both cytokine secreting cells there was no difference between N-negative and N-positive children at the follow up period.

## 4 Discussion

This study describes the long safety and the duration of the immune response in children following a heterologous vaccination schedule consisting of two doses of SOBERANA®02 followed by a third dose of SOBERANA® Plus.

Assessing long-term safety is essential to understanding the vaccine's impact on children's health, including the possibility of delayed side effects. In this study, we examine the safety of the COVID-19 vaccine in children up to 7 months after the final dose, a follow-up post-vaccination period evaluated for mRNA vaccines [16]. No adverse events of special interest, such as coagulation disorders, myocarditis, or pericarditis were reported. None of the participants withdrew from the study during the follow-up period due to post-vaccination adverse events, as has been observed in other vaccine studies with mRNA vaccine [16]. These results complement the previous safety reports of SOBERANA®02 and SOBERANA® Plus in children [5,11].

In adults aged 19–80 years, 7–8 months after the third dose there was a decrease in anti-RBD IgG levels, and in both molecular and viral neutralization titers, compared to levels observed post-third dose; but these values remained similar to or higher than those measured after the second dose [8]. This previous study did not assess immunological parameters in potential asymptomatic COVID-19 cases during the follow-up period and the positive RT-PCT individuals were excluded from the analysis. Here, the detection of SARS-CoV-2 nucleocapsid (N) protein in children's sera was included, and asymptomatic cases were not excluded but analyzed separately.

The follow-up period was epidemiologically characterized by an increase in COVID-19 cases in Havana, associated to the appearance of the omicron variant in early 2022 [17]. Of the 270 subjects considered for immunogenicity analysis 5–7 months after vaccination, 201 children (74.4 %) were positive for protein N. Among them, only 43 reported symptoms and were positive for SARS-CoV-2 by RT-PCR. This suggests a significant rate of asymptomatic infection in vaccinated children, but this was not the aim of this study.

In N-negative children, a significant decrease for all immunological variables was observed 5–7 months after vaccination compared to values after the third dose. However, as observed in adults [8], the humoral immune response was higher than after the second dose, with significant differences for % inhibition of the RBD:hACE2 interaction, molecular and viral neutralization, demonstrating the persistence of a functional antibody response. Notably, neutralizing antibodies were also detected against omicron BA.1 variant at levels 1.7 times lower than those elicited against the D614G SARS-CoV-2 variant.

Waning of the immune response has also been observed following immunization with mRNA vaccines and inactivated virus vaccines. Li et al. observed a 2.4–3.2-fold reduction of anti-RBD IgG in children aged 6–11 y/o with no evidence of prior SARS-CoV-2 infection, 5–7 months after two doses of an inactivated virus vaccine [18]. Another study in adolescents 12–17 y/o and young adults 18–19 y/o also informed a decline in the immune response (IgG anti-RBD, neutralizing and antibody-dependent cellular phagocytosis) six months after Pfizer-BioNTech mRNA vaccination. Some cross-reactivity against omicron variant was also observed, but the immune response vs. D614G was significantly higher than vs. omicron for all immunological variables at all the evaluated time points [13].

As expected, we observed that vaccinated children who were infected by SARS-CoV-2 during the follow-up period (N-protein positive) have significantly higher values for all immunological variables than children who were not infected with the virus. Prior immunity increased the humoral response 1 and 6-months after vaccination with Pfizer-BioNTech mRNA vaccine. SARS-CoV-2-recovered children 5–11 y/o showed significantly higher titers of anti-RBD IgG and neutralizing antibodies directed against wild-type or BA.2 omicron variants than SARS-CoV-2-naïve participants [15]. In the present study, the RBD-specific T-cell response remained stable six months after vaccination. The number of IFN-γ and IL-4 secreting cells was similar in individuals exposed to the SARS-CoV-2 virus (N positive) during the follow-up period and in those who were unexposed (N negative). A persistence of the anti-spike T-cell response, despite the decrease in antibody level, has been observed 3–6 months after vaccination with BNT162b2 in healthcare workers [19,20]. However, a reduction in spike-specific IFN-γ secreting cells was found after five months in healthy adolescents vaccinated with BNT162b2 [21]. Another study in children aged 5–11 y/o reported no difference in the kinetics of T subpopulations in SARS-CoV-2- naïve children 1 and 6 months after vaccination with BNT162b2, but a decrease in T-regs and CD4+ cells was detected in SARS-CoV-2 recovered children [15].

The heterologous regimen of SOBERANA® 02 followed by a third dose of SOBERANA® Plus demonstrated an effectiveness of 83.5 % (95 % CI, 82.8–84.2 %) in preventing symptomatic diseases caused by the omicron variant in children aged 3–11 years [5]. The sustained immune response, particularly the T-cell response and neutralizing antibodies against omicron BA.1 observed in this study, integrated with other immune mechanism as B and T memory may contribute to protection against COVID-19 in children [12].

Our study has some limitations. First, the sample size for T-cell immunity evaluation was small, especially when N-positive and N-negative analysis was included. Second, T-cell response was only tested against RBD from SARS-CoV-2 Wuhan variant and not against omicron variants. Third, the cross-neutralizing response was tested against omicron subvariant BA.1 but not against recently emerged omicron sub-variants. This will be the focus of a manuscript in preparation.

In conclusion, these results indicate that both the humoral and cellular immune responses generated after vaccination with the SOBERANA heterologous schedule persist at least for 5–7 months in children and adolescents aged 3–18 years, with cross-reactivity to early omicron sub-variant. With the SARS-CoV-2 virus still circulating and new variants constantly appearing, the risk of emergence of a more pathogenic strain remains likely to occur. In that scenario, studies on durability of the immune response after vaccination, and the influence of booster doses could support more effective immunization schedules.

## Author contributions

DGR, BPM, MCR and CVS conceptualized the study. RPG and YRD were clinical investigator of the trial. CVS performed the statistical analysis. RPN, LRN, DSM, YCR, SFC, ENR, OCS, BSR, THG, APD evaluated the immunological samples. AGL was responsible for monitoring the trial. SFC, YCR, DSM, MMP, YVB, VVB were responsible for vaccine development and manufacturing. DGR ans SFC drafted the manuscript. All authors critically reviewed the manuscript for important intellectual content and approved the final version.

## Funding

This work was supported by the Finlay Vaccine Institute and the National Funds for Sciences and Technology from the 10.13039/501100008807Ministry of Science, Technology and Environment (FONCI-CITMA-Cuba, contract 2020–20).

## CRediT authorship contribution statement

**Dagmar García-Rivera:** Writing – review & editing, Writing – original draft, Methodology, Conceptualization. **Rinaldo Puga-Gómez:** Investigation. **Sonsire Fernández-Castillo:** Writing – review & editing, Writing – original draft, Project administration. **Beatriz Paredes-Moreno:** Supervision, Methodology, Conceptualization. **Yariset Ricardo-Delgado:** Methodology, Investigation. **Meiby Rodríguez-González:** Supervision, Conceptualization. **Carmen Valenzuela Silva:** Writing – original draft, Software, Methodology, Formal analysis, Conceptualization. **Rocmira Pérez-Nicado:** Writing – original draft, Methodology, Investigation, Formal analysis. **Laura Rodríguez-Noda:** Investigation. **Darielys Santana-Mederos:** Project administration, Investigation. **Yanet Climent-Ruiz:** Investigation. **Enrique Noa-Romero:** Investigation. **Otto Cruz-Sui:** Investigation. **Belinda Sánchez-Ramírez:** Investigation. **Tays Hernández-García:** Investigation. **Ariel Palenzuela-Diaz:** Investigation. **Marisel Martínez-Perez:** Project administration. **Arilia García-López:** Validation, Supervision. **Yury Valdés-Balbín:** Supervision, Project administration. **Vicente G. Vérez-Bencomo:** Supervision, Project administration.

## Declaration of competing interest

The authors declare the following financial interests/personal relationships which may be considered as potential competing interests:

The authors DGR, SFC, BPM, MRG, RPN, DSM, MMP, YCR, YVB, VVB declare to be employees at Finlay Vaccine Institute. The rest of the authors declare that they have no conflict of interest.

No author received an honorarium for contributing to this paper.

## Data Availability

Data will be made available on request.
